# Calibrated scintigraphic imaging procedures improve quantitative assessment of the cardiac sympathetic nerve activity

**DOI:** 10.1038/s41598-020-78917-8

**Published:** 2020-12-14

**Authors:** Koichi Okuda, Kenichi Nakajima, Chiemi Kitamura, Yumiko Kirihara, Mitsumasa Hashimoto, Seigo Kinuya

**Affiliations:** 1 Department of Physics, Kanazawa Medical University, 1-1 Daigaku, Uchinada, Kahoku, Ishikawa 920-0293 Japan; 2 Department of Functional Imaging and Artificial Intelligence, Graduate School of Advanced Preventive Medical Sciences, Kanazawa University, 13-1 Takara-machi, Kanazawa, Ishikawa 920-8641 Japan; 3 FUJIFILM Toyama Chemical Co. Ltd., 14-1, Kyobashi 2-Chome, Chuo-Ku, Tokyo, 104-0031 Japan; 4 Department of Nuclear Medicine, Kanazawa University Hospital, 13-1 Takara-machi, Kanazawa, Ishikawa 920-8641 Japan

**Keywords:** Radionuclide imaging, Molecular imaging

## Abstract

The ^123^I-labeled meta-iodobenzylguanidine (MIBG) is an analogue of noradrenaline that can evaluate cardiac sympathetic activity in scintigraphy. Quantitative analysis of ^123^I-MIBG images has been verified in patients with heart failure and neurodegenerative diseases. However, quantitative results differ due to variations in scintigraphic imaging procedures. Here, we created and assessed the clinical feasibility of a calibration method for ^123^I-MIBG imaging. The characteristics of scintigraphic imaging systems were determined using an acrylic calibration phantom to generate a multicenter phantom imaging database. Calibration factors corresponding to the scintigraphic imaging procedures were calculated from the database and applied to a clinical study. The results of this study showed that the calibrated analysis eliminated inter-institutional differences among normal individuals. In summary, our standardization methodology for ^123^I-MIBG scintigraphy could provide the basis for improved diagnostic precision and better outcomes for patients.

## Introduction

The noradrenaline analogue ^123^I-labeled meta-iodobenzylguanidine (MIBG) allows the visualization of cardiac sympathetic nerve activity^1,2^, and cardiac scintigraphy with ^123^I-MIBG has played important roles in the diagnostic evaluation of heart failure^3–6^ and neurodegenerative diseases^7–13^. The heart-to-mediastinum ratio (HMR), calculated as ^123^I-MIBG accumulation in the heart divided by that in the mediastinum^14,15^, has been quantitatively applied to evaluate cardiac sympathetic nerve activity in ^123^I-MIBG images.

The HMR is considerably influenced by the location and size of cardiac and mediastinal regions of interest (ROI) on ^123^I-MIBG planar images. This is because ^123^I-MIBG planar image processing has not been standardized^16–19^. We developed a semi-automated method for standardizing the size and position of myocardial and mediastinal ROI to overcome this issue^20^. However, HMR variation persisted due to the characteristics of scintigraphic imaging systems such as gamma cameras and collimators, as well as the thin septa of collimators that can be easily penetrated by high-energy 529 photons emitted by ^123^I radioisotope^21,22^. This degrades the quality of ^123^I-MIBG images, and decreases the HMR^21–24^.

Consequently, we also developed a method of cross-calibrating HMR based on the performance of various collimators^25–28^ that can translate all HMR derived from various collimators and unify them as though are derived from a single collimator. We refer to this process as a method for standardizing HMR. The method is based on an acrylic chest phantom that was designed for ^123^I-MIBG planar imaging^23^. It can calibrate collimator performance differences in clinical HMR calculations that lead to standardized HMR. We validated this method in multicenter phantom studies in Japan and Europe^28–33^. The calibration phantom has been imaged using 225 and 210 collimators in Japan^26^ and Europe^33^, respectively. The findings of these studies validated the feasibility of HMR standardization using the phantom-based method. However, some minor differences in HMR have persisted^28^. Relationships between HMR and imaging conditions, and between HMR and the characteristics of gamma cameras with collimators also remain obscure.

Here, we present an improved standardization method for HMR based on combinations of gamma cameras and collimators. A multicenter phantom imaging database was created to identify the cause of HMR variations in imaging conditions, revealed that the energy-window setting for ^123^I is indispensable for robust HMR values. Moreover, this database allows the determination of mean calibration factors in combinations of gamma cameras and collimators. A clinical study showed that the standardization method with mean calibration factors is valid for patients with normal ^123^I-MIBG uptake. Our results indicate a vital role of HMR calculations in cardiac ^123^I-MIBG examinations.

## Results

### Monte Carlo simulation

**Figure 1:**
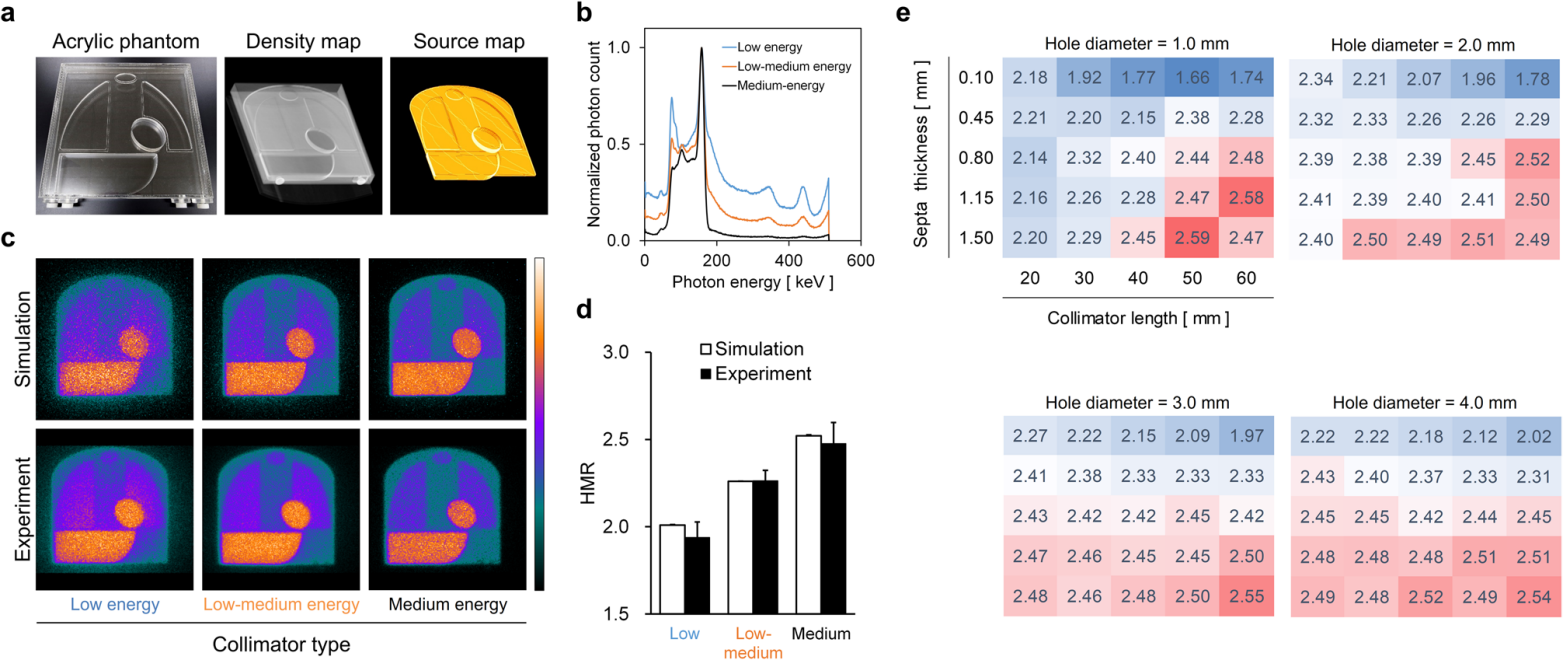
Monte Carlo simulation of ^123^I-MIBG phantom images. (**a**) Acrylic ^123^I-MIBG phantom (left), density (middle) and radioactive source (right) maps of simulation materials. (**b**) Energy spectra of ^123^I-MIBG phantom imaging in low-, low-medium, and medium-energy collimators. (**c**) Simulated (upper) and experimental (lower) phantom images generated with low- (left), low-medium (middle), and medium- (right) energy collimators. (**d**) HMR calculated from simulated and experimental phantom images using three types of collimator. Error bars represent SD of means (Student *t* tests). Simulated and experimental HMR do not significantly differ in the three collimators. (**e**) Heat maps of HMR according to collimator design. Hole diameter, septal thickness, and length of collimators ranged from 1.0 to 4.0, 0.10–1.50, and 20–60 mm, respectively. *HMR* heart-to-mediastinum count ratio.

We conducted a Monte Carlo simulation of ^123^I-MIBG phantom imaging (Fig. 1). A digital phantom was generated from an acrylic ^123^I-MIBG phantom. Density and source maps for the simulation were created from phantom images acquired by X-ray computed tomography (CT) (Fig. 1a). The energy spectra were dependent on low-energy (LE), low-medium-energy (LME), and medium-energy (ME) collimators during planar imaging (Fig. 1b). When the simulation and experimental ^123^I-MIBG phantom images were compared in terms of LE, LME, and ME collimators, image blurring due to 529-keV high-energy photon was visualized under both conditions with the LE collimator (Fig. 1c). The HMR in the three collimators did not significantly differ between the simulation and experimental conditions (Fig. 1d). Septal thickness, collimator length, and diameter of the collimator hole were major components of HMR variation (Fig. 1e).

### HMR variation under acquisition conditions

**Figure 2:**
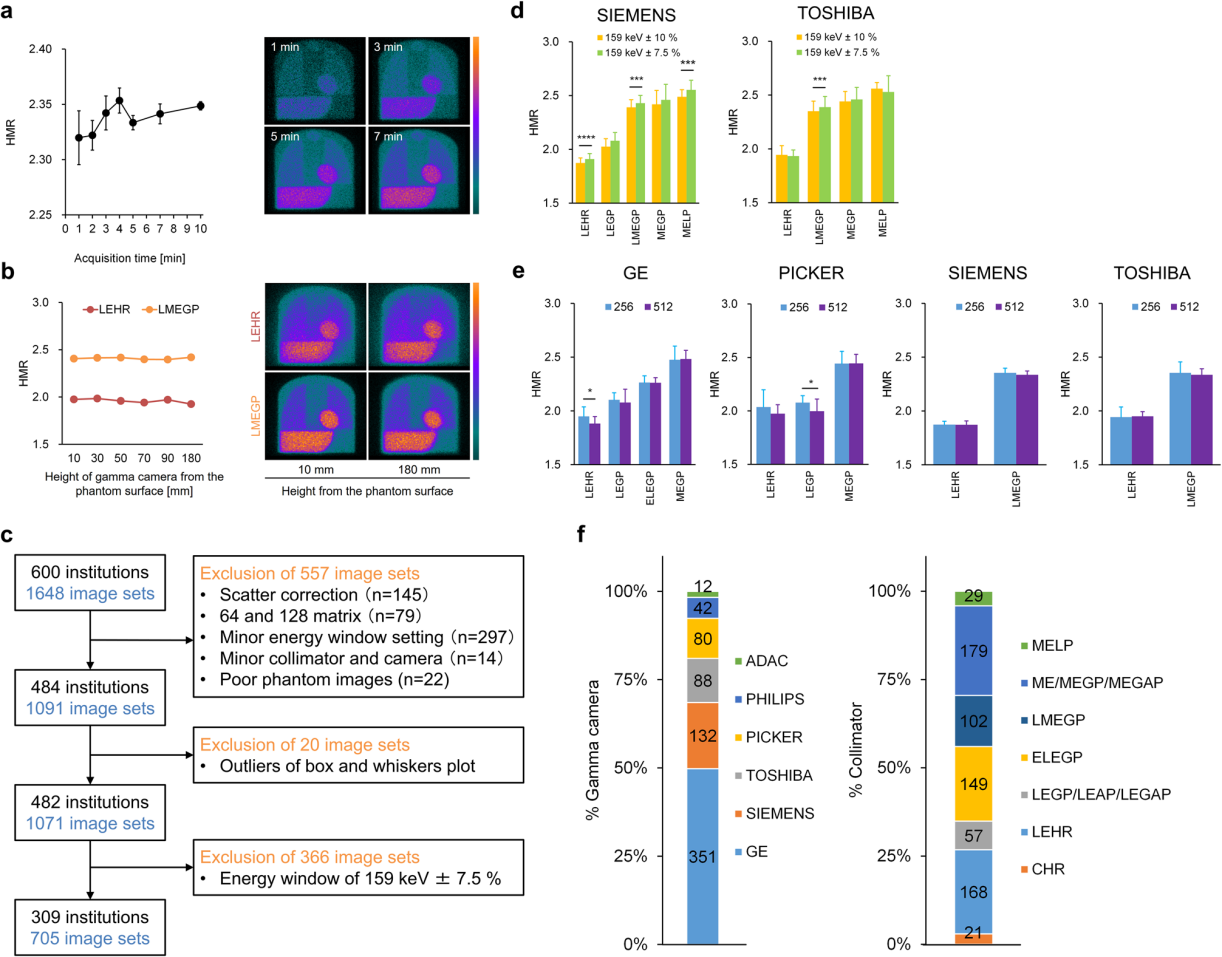
^123^I-MIBG imaging characteristics in multicenter phantom study. (**a**) HMR does not significantly differ among 1-, 2-, 3-, 4-, 5-, 7-, and 10-min image acquisitions. Images were acquired from phantom in 1-, 3-, 5-, and 7-min. (**b**) Relationships between HMR and gamma cameras located 10, 30, 50, 70, 90, and 180 mm from phantom surface. Phantom images derived using LEHR and LMEGP collimators located 10- and 180-mm above phantom surface. (**c**) Eligible phantom image datasets based on selection criteria in multicenter phantom imaging study. (**d**) Comparison of HMR at energy windows of 159 keV ± 10% and 159 keV ± 7.5% in equipment from two vendors. (**e**) Comparison of HMR with matrices of 256 and 512 in equipment from four vendors. (**f**) Gamma cameras manufactured by six vendors (left) and seven types of collimators (right) were included in multicenter phantom image datasets. Error bars are SD of mean. *P < 0.05, ***P < 0.001, and ****P < 0.0001. Paired *t*-test for each comparison in (**a**), Wilcoxon singed rank test in (**d**), and Student *t* test in (**e**). *CHR* cardiac high-resolution, *ELEGP* extended low-energy general-purpose, *HMR* heart-to-mediastinum count ratio, *LEAP* low-energy all-purpose, *LEGAP* low-energy general-all-purpose, *LEGP* low-energy general-purpose, *LEHR* low-energy high-resolution, *LMEGP* low-medium-energy general-purpose, *ME* medium-energy, *MEGAP* medium-energy general-all-purpose, *MEGP* medium-energy general-purpose, *MELP* ME low-penetration.

We confirmed the HMR in ^123^I-MIBG planar images obtained over periods of 1–10 min. Although mean HMR did not significantly differ among acquisition periods of 1, 2, 3, 4, 5, 7, and 10 min (2.32 ± 0.063, 2.32 ± 0.035, 2.34 ± 0.040, 2.35 ± 0.029, 2.33 ± 0.017, 2.34 ± 0.024, and 2.35 ± 0.010, respectively), the standard deviations (SD) of the HMR gradually decreased over longer acquisition periods (Fig. 2a). The quality of phantom images seemed most stable during acquisition for 5 and 7 min. The HMR were also stable at any gamma camera position from the phantom surface (Fig. 2b). Image quality was degraded in both LE high-resolution (LEHR) and LME general-purpose (LMEGP) collimators when the distance from the phantom surface was increased. The HMR was higher when images were acquired at the energy windows of 159 keV ± 7.5% than at 159 keV ± 10%, indicating that the setting of primary energy window of ^123^I affected the HMR (Fig. 2d). The HMR values with 256 and 512 matrices did not significantly differ except under two imaging conditions (Fig. 2e).

### Multicenter ^123^I-MIBG phantom image database

Among 1648 phantom image sets from 600 institutions accumulated in Japan between February 2009 and April 2017, 705 were eligible as multicenter phantom data (Fig. 2c). The imaging conditions of the ^123^I-MIBG phantom database were as follows: imaging matrices, 256 and 512; median pixel size, 2.21 (IQR 1.47–2.26) mm; and median acquisition time, 300 (60–900) s. Figure 2f shows the numbers and ratios (%) of gamma cameras and collimators included in the multicenter ^123^I-MIBG phantom image database.

### Conversion coefficient of gamma camera-collimator combinations

**Figure 3:**
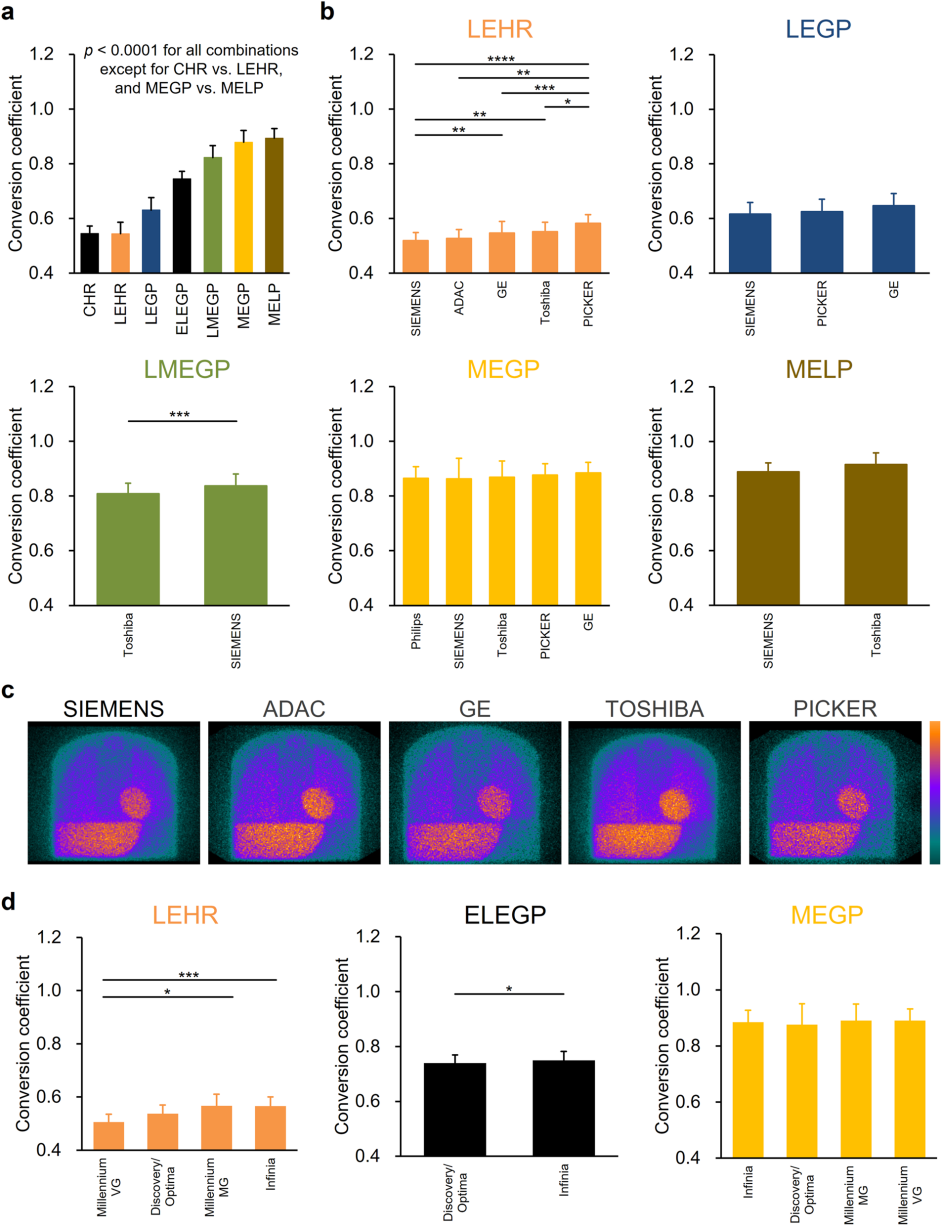
Conversion coefficients in multicenter ^123^I-MIBG phantom study. (**a**) Mean conversion coefficients in CHR, LEHR, LEGP/LEAP/LEGAP, ELEGP, LMEGP, ME/MEGP/MEGAP, and MELP collimator categories. (**b**) Conversion coefficients obtained using following collimators from LEHR, LEGP, LMEGP, MEGP, and MELP from 5, 3, 2, 5, and 2 vendors, respectively. (**c**) ^123^I-MIBG phantom image quality obtained using LEHR collimator from 5 vendors. (**d**) Conversion coefficients obtained from 4, 2, and 4 GE gamma cameras with LEHR, ELEGP, and MEGP collimators, respectively. Error bars are SD of means. *P < 0.05, ^**^P < 0.01, ^***^P < 0.001, and ****P < 0.0001. Tukey–Kramer and Student *t*-tests in **a**, **b** and **d**. *CHR* cardiac high-resolution, *ELEGP* extended low-energy general-purpose, *LEGP* low-energy general-purpose, *LEHR* low-energy high-resolution, *LMEGP* low-medium-energy general-purpose, *MEGP* medium-energy general-purpose, *MELP* ME low-penetration.

We examined conversion coefficients in the following collimator categories (Fig. 3a): cardiac high-resolution (CHR), low-energy high-resolution (LEHR), low-energy general-purpose (LEGP), low-energy all-purpose (LEAP), low-energy general-all-purpose (LEGAP), extended low-energy general-purpose (ELEGP), low-medium-energy general-purpose (LMEGP,) medium-energy (ME), medium-energy general-purpose (MEGP), medium-energy general-all-purpose (MEGAP), and medium-energy low-penetration (MELP). The mean conversion coefficients were 0.545 ± 0.0268 for CHR (n = 21); 0.545 ± 0.0414 for LEHR (n = 167); 0.631 ± 0.0455 for LEGP, LEAP, and LEGAP (n = 57); 0.745 ± 0.0268 for ELEGP (n = 149); 0.823 ± 0.0437 for LMEGP (n = 102); 0.879 ± 0.0429 for ME, MEGP, and MEGAP (n = 179), and 0.894 ± 0.0349 for MELP (n = 29). These conversion coefficients evaluated in individual LEGP, MEGP, and MELP collimators did not significantly differ among vendors (Fig. 3b). Conversion coefficients were independent of manufacturers, being 0.519 ± 0.0296 for Siemens (n = 42), 0.527 ± 0.0326 for ADAC (n = 6), 0.546 ± 0.0433 for GE (n = 67), 0.552 ± 0.0347 for Toshiba (n = 24), and 0.583 ± 0.0315 for Picker (n = 25) in LEHR collimators; and 0.808 ± 0.0388 for Toshiba (n = 49) and 0.836 ± 0.0440 for Siemens (n = 53) in LMEGP collimators. Count statistics widely varied in the mediastinum determined from ^123^I-MIBG phantom images derived using LEHR collimators (Fig. 3c). When GE gamma cameras were combined with LEHR, ELEGP, and MEGP collimators, conversion coefficients significantly differed among the Millennium VG (0.506 ± 0.0318, n = 11), Millennium MG (0.567 ± 0.0480, n = 10) and Infinia (0.566 ± 0.0342, n = 26) with LEHR collimator; and between Discovery/Optima (0.740 ± 0.0262, n = 70) and Infinia (0.750 ± 0.0268, n = 79) with ELEGP collimator (Fig. 3d). Mean conversion coefficients of combinations of gamma cameras with collimators were determined using the multicenter ^123^I-MIBG phantom image database (Table 1).

**Table 1:** Average multicenter conversion coefficients for combinations of gamma cameras and collimators obtained from 705 image sets.

	CHR	LEHR	LEGP	ELEGP	LMEGP	MEGP	MELP
**GE**							
Discovery, Optima	–	0.54	–	0.73	–	0.88	–
Infinia	–	0.56	–	0.75	–	0.88	–
Millennium MG	–	0.59	0.65	–	–	0.89	–
Millennium VG	–	0.50	0.64	–	–	0.89	–
**PHILIPS**							
BrightView	0.53	–	–	–	–	0.84	–
**PICKER**							
PRISM	–	0.58	0.62	–	–	0.87	–
**SIEMENS**							
e.cam, Symbia	–	0.51	–	–	0.84	0.85	0.88
Evo Excel, Intevo	–	0.51	–	–	0.81		0.88
**TOSHIBA**							
e.cam, Symbia	–	0.55	–	–	0.81	0.87	0.90

*CHR* cardiac high-resolution, *ELEGP* extended low-energy general-purpose, *LEGP* low-energy general-purpose, *LEHR* low-energy high-resolution, *LMEGP* low-medium-energy general-purpose, *MEGP* medium-energy general-purpose, *MELP* ME low-penetration.

To confirm the flexibility of conversion coefficients to account for the variation in image acquisitions, we calculated average multicenter conversion coefficients from the phantom image database consisted of 1459 phantom image sets and compared these conversion coefficients and those from the database consisted of 705 image sets in Supplementary Table 1. The average conversion coefficients were statistically equivalent between two databases in CHR, LEHR, LEGP, ELEGP, LMEGP, and MEGP collimators except for MELP collimator. Consequently, we additionally determined the average multicenter conversion coefficients of combinations of gamma cameras with collimators in 1459 phantom image sets (Supplementary Table 2).

### Clinical application of the method for standardizing ^123^I-MIBG scintigraphy

**Figure 4:**
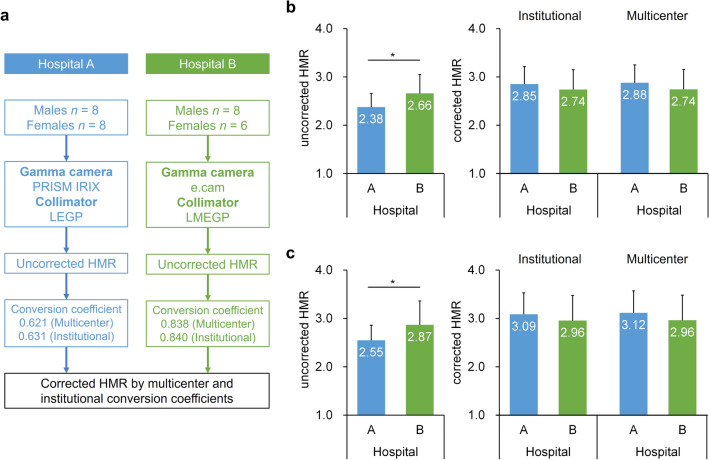
Clinical implementation of HMR standardization using conversion coefficients. (**a**) HMR standardization using institutional and multicenter conversion coefficients. (**b**) Uncorrected and corrected HMR in early ^123^I-MIBG images. (**c**) Uncorrected and corrected HMR in delayed ^123^I-MIBG images. Error bars are SD of means. *P < 0.05. Student *t*-test and paired *t*-test in (**b**) and (**c**). *HMR* heart-to-mediastinum count ratio, *LEGP* low-energy general-purpose, *LMEGP* low-medium-energy general-purpose.

Normal HMR values of two hospitals were evaluated with or without standardization (Fig. 4a). The HMR for early and delayed ^123^I-MIBG scintigraphic images were corrected with institutional and multicenter conversion coefficients. The institutional conversion coefficients were 0.631 and 0.840 for hospitals A and B, respectively. The multicenter conversion coefficients were 0.621 and 0.838 for hospitals A and B, respectively. Although the averaged normal values in hospitals A and B significantly differed before standardization, the standardized normal values did not significantly differ between these hospitals in both early and delayed ^123^I-MIBG images (Fig. 4b,c). Furthermore, HMR corrected with institutional and multicenter conversion coefficients did not significantly differ.

To evaluate the accuracy of the calibration factor, we calculated net reclassification improvement^34^ (NRI) in normal subjects and patients with heart failure. Of the 12 patients (24 image sets) who were diagnosed with heart failure, classification was improved in four images when the calibration factor was applied to H/M ratio. However, of the 21 normal subjects (42 image sets), classification was only worsened in one image. The NRI in all subjects showed 14.3% (*p* = 0.099). Uncorrected and corrected HMR values for individual heart failure patients are shown in Supplementary Table 3.

## Discussion

The major findings of the present simulation study were that collimator design, including collimator length, hole diameter, and septal thickness, affect ^123^I-MIBG image quality and HMR. The phantom studies revealed that the energy-window setting for ^123^I is an important factor for reducing HMR variation. However, HMR variations due to acquisition time, matrix size, and distance between gamma camera and phantom surfaces were limited. The conversion coefficients represented the characteristics of gamma cameras and collimators in the multicenter phantom study, and differed among manufacturers of collimators even those with the same name. Moreover, conversion coefficients significantly differed in some combinations of gamma cameras with collimators, even within individual manufacturers. In the clinical validation study, the standardization methodology yielded equivalent normal HMR values corrected with both institutional and multicenter conversion coefficients.

Monte Carlo simulation provided reasonable ^123^I-MIBG phantom planar images even considering the effects of the 529 keV photons added to the 159 keV photons. Although the fractions of 529 and159 keV photons of ^123^I were 1.39% and 83.3%, respectively, the high-energy photons hampered quantitative analysis of HMR and degrade ^123^I-MIBG planar image quality. Since these 529 keV photons easily penetrated thin collimator septa, a peak appeared in the energy spectrum with the LEHR collimator. Photons that penetrated the septum or scattered, also degraded ^123^I-MIBG planar image quality and reduced the HMR. In addition to septal thickness, collimator length and hole diameter are also important components that determine both ^123^I-MIBG image quality and HMR. Thick collimator septa, small hole diameters, and long collimators are most appropriate. Considering these effects of 529 keV photons, the MEGP collimator is adequate for ^123^I-MIBG imaging.

We previously determined conversion coefficients for several collimator groups in 225 experiments at 84 institutions^26^. A comparison of mean conversion coefficients between the present and previous phantom studies revealed the following: 0.55 ± 0.027 (n = 21) vs. 0.55 ± 0.02 (n = 9) for CHR (*p* = n.s.); 0.55 ± 0.041 (n = 167) vs. 0.55 ± 0.05 (n = 73) for LEHR (*p* = n.s.); 0.63 ± 0.046 (n = 57) vs. 0.65 ± 0.04 (n = 25) for LEGP, LEAP, and LEGAP (*p* = n.s.); 0.75 ± 0.027 (n = 149) vs. 0.75 ± 0.03 (n = 14) for ELEGP (*p* = n.s.); 0.82 ± 0.044 (n = 102) vs. 0.83 ± 0.05 (n = 46) for LMEGP (*p* = n.s.); 0.88 ± 0.043 (n = 179) vs. 0.88 ± 0.05 (n = 40) for ME, MEGP, and MEGAP (*p* = n.s.), and 0.89 ± 0.035 (n = 29) vs. 0.95 ± 0.04 (n = 14) for MELP (*p* < 0.0001), respectively. These results showed that only the MELP collimators significantly differed. The MELP collimators were manufactured by Toshiba Medical Systems Corporation and Siemens Healthineers. The present findings showed that although the conversion coefficients were equivalent between the two vendors (Fig. 3b), they were affected by the energy window setting of ^123^I (Fig. 2d). We applied a single energy window setting in the present study, whereas windows were set at 159 keV ± 10% and 159 keV ± 7.5% in the previous study. In Supplementary Table 1, when we compared two imaging databases acquired with single and various energy window settings, the average values of conversion coefficients were significantly different in for the MELP collimator condition.

Imaging conditions need standardization in addition to HMR for ^123^I-MIBG image acquisition. A tremendous amount of data regarding imaging protocols has been accumulated in the multicenter phantom image database with respect to the imaging matrix, energy window setting of ^123^I, and acquisition time. Moreover, 145 image datasets scatter-corrected using ^123^I dual-^35,36^ and triple-energy^23,37^ windows were included in the phantom database. Since the clinical usage of ^123^I-MIBG was approved in 1992 in Japan, many studies have investigated the HMR quantitation^23,38–41^, which has led to a wide variety of imaging conditions and correction methods. In addition, ^123^I-MIBG phantom experiments conducted in the Netherlands, Belgium, the UK, Austria, and Italy^28–30,32,33^ have also generated a considerable amount of data. Our phantom-based standardization methodology allows international comparisons of HMR.

Our study has several limitations. We used an institutional ^123^I-MIBG imaging procedure for the phantom scans. Therefore, the imaging procedure was not unified in the multicenter phantom study. However, eligible phantom image sets were selected according to the selection criteria of the multicenter ^123^I-MIBG phantom image database (Fig. 2c). Although we provided multicenter conversion coefficients to standardize HMR, they could only be used at the energy-window setting of 159 keV ± 10%. Since the number of conversion coefficients for the energy-window setting of 159 keV ± 7.5% was limited (Supplementary Table 4), additional multicenter ^123^I-MIBG phantom imaging studies are needed to accumulate conversion coefficients for this setting. The clinical validation study confirmed the feasibility of our method only for patients with normal ^123^I-MIBG distribution. A multicenter clinical trial should be conducted using institutional and multicenter conversion coefficients.

In conclusion, our standardization methodology for ^123^I-MIBG scintigraphy allowed determination of the characteristics of gamma cameras and collimator combinations in the multicenter phantom study. The clinical validation study showed that normal HMR derived from two different institutions did not significantly differ after standardization.

## Material and methods

### Quantitative analysis in ^123^I-MIBG imaging

The HMR was used to calculate cardiac ^123^I-MIBG accumulation in planar images as cardiac ^123^I-MIBG uptake divided by background of ^123^I-MIBG distribution using ROI positioned over the heart and over the upper mediastinum^14^. Fully and semi-automated ROI setting algorithms were applied to the phantom and clinical studies^20^, respectively. The HMR were automatically calculated using both algorithms.

### Calibration phantom for planar ^123^I-MIBG imaging

A flat, polymethyl methacrylate phantom (Taisei Medical, Co. Ltd, Osaka, Japan) was developed to calibrate HMR under various imaging conditions with collimators^23,33^ (Fig. 1a). The volume (width × depth × height) of this phantom is 380 × 380 × 50 mm^3^, and it can mimic planar ^123^I-MIBG distribution in the heart, mediastinum, liver, lungs, and thyroid gland. Anterior and posterior planar ^123^I-MIBG images were acquired from both sides of the phantom. The designated HMR of the anterior and posterior views were 2.60 and 3.50, respectively. Details of the phantom design have been published elsewhere^23^.

### Calibration factor for gamma camera and collimator system

The calibration factor was calculated from the HMR derived from anterior (HMR_Ant_) and posterior (HMR_Post_) planar ^123^I-MIBG phantom images using dedicated software and is defined as a conversion coefficient calculated as:

$$ {\text{Conversion}}\;{\text{coefficient}} = {{\left( {{{\left( {{\text{HMR}}_{{{\text{Ant}}}} + {\text{HMR}}_{{{\text{Post}}}} } \right)} \mathord{\left/ {\vphantom {{\left( {{\text{HMR}}_{{{\text{Ant}}}} + {\text{HMR}}_{{{\text{Post}}}} } \right)} {{2} - {1}}}} \right. \kern-\nulldelimiterspace} {{2} - {1}}}} \right)} \mathord{\left/ {\vphantom {{\left( {{{\left( {{\text{HMR}}_{{{\text{Ant}}}} + {\text{HMR}}_{{{\text{Post}}}} } \right)} \mathord{\left/ {\vphantom {{\left( {{\text{HMR}}_{{{\text{Ant}}}} + {\text{HMR}}_{{{\text{Post}}}} } \right)} {{2} - {1}}}} \right. \kern-\nulldelimiterspace} {{2} - {1}}}} \right)} {\left( {{{\left( {{2}.{6}0 + {3}.{5}0} \right)} \mathord{\left/ {\vphantom {{\left( {{2}.{6}0 + {3}.{5}0} \right)} {{2} - {1}}}} \right. \kern-\nulldelimiterspace} {{2} - {1}}}} \right)}}} \right. \kern-\nulldelimiterspace} {\left( {{{\left( {{2}.{6}0 + {3}.{5}0} \right)} \mathord{\left/ {\vphantom {{\left( {{2}.{6}0 + {3}.{5}0} \right)} {{2} - {1}}}} \right. \kern-\nulldelimiterspace} {{2} - {1}}}} \right)}}, $$

where, 2.60 and 3.50 are the respective designated HMR in anterior and posterior views of the calibration phantom. An institutional conversion coefficient (CC_i_) was derived from the anterior and posterior phantom images after image acquisition under institutional ^123^I-MIBG planar imaging conditions.

### Conversion to standardized HMR using the calibration factor

Since the European Association Nuclear Medicine and the European Council of Nuclear Cardiology have proposed using MEGP collimators for^123^I-MIBG imaging^16^, all HMR were converted into that for a MEGP collimator. A standardized conversion coefficient (CC_std_) has already been defined as 0.88^26^. The CC_i_ and CC_std_ allow for the conversion of all institutional HMR (HMR_i_) into standardized HMR (HMR_std_) using the equation^26^:

$$ {\text{HMR}}_{{{\text{std}}}} = {\text{CC}}_{{{\text{std}}}} /{\text{CC}}_{{\text{i}}} \times \left( {{\text{HMR}}_{{\text{i}}} - {1}} \right) + {1}. $$

### Monte Carlo simulation for ^123^I-MIBG imaging

A digital phantom image was created from the acrylic calibration phantom image acquired using X-ray CT. Density and source maps of the phantom were generated for the following simulations. The simulation of imaging nuclear detectors (SIMIND; Lund University, Lund, Sweden) Monte Carlo program^42^ allowed ^123^I-MIBG planar imaging simulations using various types of collimators. Combinations of the following collimator conditions were examined: collimator hole diameters of 1, 2, 3, 4, and 5 mm; septal thicknesses of 0.10, 0.45, 0.80, 1.15, and 1.50 mm, and collimator lengths of 20, 30, 40, 50, and 60 mm. We generated ^123^I-MIBG planar images using a total of 8.65 × 10^8^ photons. The number of detected photons ranged from 418 to 15,321 per second. Planar MIBG imaging was simulated with 256 × 256 matrices, and the energy window of ^123^I was set at 159 keV ± 7.5%.

### HMR variations during various acquisition periods

Anterior MIBG planar images were acquired using a dual-head gamma camera (e.cam; Toshiba Medical Systems, Tokyo, Japan) and an LMEGP collimator over periods of 1, 2, 3, 4, 5, 7, and 10 min from the phantom containing 55.5 MBq of ^123^I-MIBG. Five image datasets were acquired during each period. Planar imaging was conducted with a 256 × 256 matrix, and a pixel size of 1.65 mm. A photopeak window of ^123^I was centered at 159 keV with a 15% energy window. This study proceeded at Narita Memorial Hospital, Aichi, Japan.

### HMR variations according to distance between gamma camera location and phantom surface

Anterior planar images were acquired from the phantom containing 55.5 MBq of ^123^I-MIBG. The phantom was equipped with a Symbia T6 dual-head gamma camera (Siemens Healthineers, Erlangen, Germany) with LEHR and LMEGP collimators. The gamma camera positions were set at 10, 30, 50, 70, 90, and 180 mm from the phantom surface for both LEHR and LMEGP collimators. The number of acquired counts was consistently 1.0 × 10^6^. Planar imaging proceeded with a 256 × 256 matrix, and 2.40-mm pixels. The photopeak window of ^123^I was centered at 159 keV with a 20% energy window. This study proceeded at Kanazawa University Hospital, Kanazawa, Japan.

### Multicenter ^123^I-MIBG phantom image database

We accumulated 1648 phantom image sets from 600 institutions in Japan between February 2009 and April 2017. The six gamma camera manufacturers selected for this database were ADAC Laboratories (Milpitas, CA, USA), GE Healthcare (Waukesha, WI, USA), Philips Medical system (Milpitas, CA, USA), Picker Corporation (Cleveland, OH, USA), Toshiba Medical Systems Corporation, and Siemens Healthineers. We excluded 145 phantom image datasets for scatter correction of the ^123^I dual- and triple-energy windows and 79 others acquired with 64 and 128 matrices. We excluded 297 minor conditions of the energy window setting (keV) for ^123^I-MIBG image acquisition as follows: 154 ± 10% (n = 4), 155 ± 10% (n = 4), 156 ± 7.5% (n = 1), 156 ± 10% (n = 32), 157 ± 10% (n = 28), 158 ± 10% (n = 101), 158 ± 10.5% (n = 1), 158 ± 12% (n = 2), 158 k ± 7% (n = 1), 158 ± 7.5% (n = 23), 159 ± 10.5% (n = 3), 159 ± 12% (n = 1), 159 ± 6.3% (n = 2), 159 ± 8% (n = 1), 159 ± 9% (n = 1), 160 ± 10% (n = 80), 160 ± 7.5% (n = 8), and missing data (n = 4). We excluded the following 14 minor collimators and gamma cameras: ^123^I (n = 1), Cardiac (n = 2), LELP (n = 1), LPHR (n = 1), and MEDIUM (n = 2) manufactured by Siemens; high energy (HE) GP (n = 1) by GE; Cardio (n = 1), and MEHR (n = 3) by Toshiba; LE ultra-high resolution (n = 1) by Picker; and RC-1500I gamma camera with LEGP collimator (n = 1) by Hitachi Medico Corporation, Chiba, Japan. Twenty-two failed phantom experiments were excluded. Mean conversion coefficients were computed for phantom images classified according to collimator groups as CHR; LEHR; LEGP, LEAP, and LEGAP; ELEGP; LMEGP; ME, MEGP, and MEGAP; and MELP. Twenty phantom image datasets were excluded due to outliers of the mean conversion coefficients based on box and whisker plots. We finally excluded 366 images acquired with the energy set at 159 keV ± 7.5%.

### HMR variation according to energy window

The HMR values obtained with the energy window setting of 159 keV ± 10% and 159 keV ± 7.5% were compared in the multicenter phantom image database comprising 1071 image datasets from 482 institutions. Since the number of image datasets was insufficient for comparisons of energy window settings of 159 keV ± 10% and 159 keV ± 7.5% in ADAC (12 vs. 0, respectively), GE (351 vs. 0, respectively), Philips (42 vs. 2, respectively), and Picker (80 vs. 3, respectively), we compared the datasets from Siemens (132 vs. 227, respectively) and Toshiba (88 vs. 134, respectively).

### HMR variation according to imaging matrix

The HMR from 256 and 512 matrices were compared in the multicenter phantom image database that comprised 705 image datasets from 309 institutions obtained with an energy window setting of 159 keV ± 10%. The image datasets from GE (n = 351), Picker (n = 80), Siemens (n = 132) and Toshiba (n = 88) were compared.

### Conversion coefficient for combinations of gamma cameras and collimators

Based on the multicenter ^123^I-MIBG phantom image database with the image selection criteria, mean conversion coefficients for combinations of gamma cameras and collimators were determined using 705 image sets from 309 institutions. The 9 types of gamma cameras were Discovery/Optima (n = 121), Infinia (n = 151), Millennium MG (n = 33), and Millennium VG (n = 33) manufactured by GE; BrightView (n = 36) by Philips; PRISM (n = 73) by Picker; e.cam/Symbia (n = 110) and EvoExcel/IntevoExcel (n = 12) by Siemens, and e.cam/Symbia (n = 79) by Toshiba. Additional multicenter ^123^I-MIBG phantom image datasets for the EvoExcel/IntevoExcel system were accumulated due to the absence of these image datasets in the phantom database. The number of additional image datasets was 27 from 14 institutions.

When the multicenter ^123^I-MIBG phantom images were selected based on two imaging conditions of whole energy-window setting and imaging matrice, mean conversion coefficients for combinations of gamma cameras and collimators were determined using 1,459 image sets from 593 institutions (Supplementary Fig. 1). The 12 types of gamma cameras were Forte (n = 40) manufactured by ADAC; Discovery/Optima (n = 128), Infinia (n = 173), Millennium MG (n = 34), and Millennium VG (n = 51) manufactured by GE; BrightView (n = 95) by Philips; PRISM (n = 92) by Picker; e.cam/Symbia (n = 425) and EvoExcel/IntevoExcel (n = 34) by Siemens, and e.cam/Symbia (n = 298), GCA 7100/7200 (n = 26), and GCA 9300 (n = 23) by Toshiba.

### Clinical validation image dataset

We applied the calibration method of HMR to an anonymized clinical image dataset. The Japanese Society of Nuclear Medicine working group (JSNM-WG) activity collected planar images from patients who were determined as normal cardiac ^123^I-MIBG uptake in 2007 and 2015^43–45^. All personal information of ^123^I-MIBG images was excluded and anonymized ^123^I-MIBG images formatted with digital imaging and communications in medicine were provided as a research database. We obtained the permission for the secondary use of the databases as a research purpose in accordance with JSNM-WG regulation. Details of the patient characteristics have been published elsewhere^43^. In the anonymized clinical image dataset, male and female (n = 8 each) ^123^I-MIBG images were collected from hospital A, and eight male and six female images were collected from hospital B (Fig. 4a). The gamma cameras and collimators were PRISM IRIX and LEGP manufactured by Shimadzu (Picker Corp., Cleveland, Ohio, USA/Shimadzu Corp., Kyoto, Japan) at hospital A, respectively, and e.cam and LMEGP manufactured by Siemens at hospital B, respectively. The acquisition time, imaging matrix, and energy-window setting for ^123^I were 5 min, 256, and 159 keV ± 10%, respectively at both hospitals. Early and delayed planar images were acquired at 15 min and 4 h after injecting ^123^I-MIBG in both hospitals, respectively. The institutional conversion coefficients were obtained from ^123^I-MIBG phantom scans at each hospital. Mean multicenter conversion coefficients matching the combinations of gamma cameras and collimators at hospitals A and B were calculated from the ^123^I-MIBG phantom image database.

For the calculation of net reclassification improvement with the standardization procedure in clinical subjects, we used clinical image datasets collected from the hospital A (16 subjects, 32 images for early and delayed conditions) and Kanazawa University Hospital, Kanazawa, Japan (17 subjects, 34 images). Regarding clinical ^123^I-MIBG imaging condition in Kanazawa University Hospital, the acquisition time, imaging matrix, and energy-window setting for ^123^I were 5 min, 256, and 159 keV ± 10%, respectively. Early and delayed planar images using an LMEGP collimator were acquired at 20 min and 3 h after injecting ^123^I-MIBG. Of the 33 patients, 12 patients were diagnosed with heart failure, and 21 subjects were diagnosed with a normal heart. The reclassification table was generated to compare standardized HMR values using multicenter conversion coefficients with uncorrected HMR values. These HMR values were classified into two patient groups using the thresholds of 2.17 and 2.49 that were determined by the receiver operating characteristic analysis in unstandardized and standardized conditions, respectively.

### Statistical analysis

All continuous values are expressed as means ± SD. The Shapiro–Wilk testing for the evaluation of normality was performed in the continuous dataset. Differences in continuous variables were analyzed using Student t-tests and Wilcoxon singed rank tests. Multiple comparisons of continuous variables were assessed using Tukey–Kramer tests. Differences in paired continuous data were analyzed using paired t-tests. All statistical tests were two-tailed, and values with p < 0.05 were considered significant. All data were statistically analyzed using JMP version 11.2.1 (SAS Institute Inc., Cary, NC, USA).

## Supplementary Information

Supplementary Information.

## Data Availability

The multicenter phantom data that support the findings of this study are available from the corresponding authors (KO and KN) upon reasonable request. Regarding the clinical normal database for ^123^I-MIBG scintigraphy, the planar ^123^I-MIBG imaging data that support the findings of this study are available from the corresponding author (KN) upon reasonable request.

## References

[1] Wieland DM (1981). Myocardial imaging with a radioiodinated norepinephrine storage analog. J. Nucl. Med..

[2] Schofer J, Spielmann R, Schuchert A, Weber K, Schluter M (1988). Iodine-123 meta-iodobenzylguanidine scintigraphy: A noninvasive method to demonstrate myocardial adrenergic nervous system disintegrity in patients with idiopathic dilated cardiomyopathy. J. Am. Coll. Cardiol..

[3] Jacobson AF (2010). Myocardial iodine-123 meta-iodobenzylguanidine imaging and cardiac events in heart failure. Results of the prospective ADMIRE-HF (AdreView Myocardial Imaging for Risk Evaluation in Heart Failure) study. J. Am. Coll. Cardiol..

[4] Nakata T (2013). A pooled analysis of multicenter cohort studies of (123)I-mIBG imaging of sympathetic innervation for assessment of long-term prognosis in heart failure. JACC Cardiovasc. Imaging.

[5] Verschure DO (2014). For what endpoint does myocardial 123I-MIBG scintigraphy have the greatest prognostic value in patients with chronic heart failure? Results of a pooled individual patient data meta-analysis. Eur. Heart J. Cardiovasc. Imaging.

[6] Travin MI, Matsunari I, Thomas GS, Nakajima K, Yoshinaga K (2019). How do we establish cardiac sympathetic nervous system imaging with ^123^I-*m*IBG in clinical practice? Perspectives and lessons from Japan and the US. Ann. Nuclear Cardiol..

[7] Orimo S, Suzuki M, Inaba A, Mizusawa H (2012). 123I-MIBG myocardial scintigraphy for differentiating Parkinson's disease from other neurodegenerative Parkinsonism: A systematic review and meta-analysis. Parkinsonism Relat. Disord..

[8] Orimo S, Ozawa E, Nakade S, Sugimoto T, Mizusawa H (1999). (123)I-metaiodobenzylguanidine myocardial scintigraphy in Parkinson's disease. J. Neurol. Neurosurg. Psychiatry.

[9] Treglia G (2012). MIBG scintigraphy in differential diagnosis of Parkinsonism: A meta-analysis. Clin. Autonomic Res. Off. J. Clin. Autonomic Res. Soc..

[10] Yoshita M (2006). Value of 123I-MIBG radioactivity in the differential diagnosis of DLB from AD. Neurology.

[11] Yoshita M (2015). Diagnostic accuracy of 123I-meta-iodobenzylguanidine myocardial scintigraphy in dementia with Lewy bodies: A multicenter study. PLoS ONE.

[12] Komatsu J (2018). (123)I-MIBG myocardial scintigraphy for the diagnosis of DLB: A multicentre 3-year follow-up study. J. Neurol. Neurosurg. Psychiatry.

[13] Yamada M (2020). Diagnostic criteria for dementia with Lewy bodies: Updates and future directions. J. Mov. Disord..

[14] Merlet P (1992). Prognostic value of cardiac metaiodobenzylguanidine imaging in patients with heart failure. J. Nucl. Med..

[15] Veltman CE (2012). Reproducibility of planar (123)I-meta-iodobenzylguanidine (MIBG) myocardial scintigraphy in patients with heart failure. Eur. J. Nucl. Med. Mol. Imaging.

[16] Flotats A (2010). Proposal for standardization of 123I-metaiodobenzylguanidine (MIBG) cardiac sympathetic imaging by the EANM Cardiovascular Committee and the European Council of Nuclear Cardiology. Eur. J. Nucl. Med. Mol. Imaging.

[17] van der Veen L, Scholte A, Stokkel M (2010). Mathematical methods to determine quantitative parameters of myocardial 123I-MIBG studies: A review of the literature. Nucl. Med. Commun..

[18] Somsen GA, Verberne HJ, Fleury E, Righetti A (2004). Normal values and within-subject variability of cardiac I-123 MIBG scintigraphy in healthy individuals: implications for clinical studies. J. Nucl. Cardiol..

[19] Nakajima K, Taki J, Tonami N, Hisada K (1994). Decreased 123I-MIBG uptake and increased clearance in various cardiac diseases. Nucl. Med. Commun..

[20] Okuda K (2011). Semi-automated algorithm for calculating heart-to-mediastinum ratio in cardiac Iodine-123 MIBG imaging. J. Nucl. Cardiol..

[21] Dobbeleir AA, Hambye AS, Franken PR (1999). Influence of high-energy photons on the spectrum of iodine-123 with low- and medium-energy collimators: consequences for imaging with 123I-labelled compounds in clinical practice. Eur. J. Nucl. Med..

[22] Inoue Y (2003). Effect of collimator choice on quantitative assessment of cardiac iodine 123 MIBG uptake. J. Nucl. Cardiol..

[23] Nakajima K (2007). Correction of iodine-123-labeled meta-iodobenzylguanidine uptake with multi-window methods for standardization of the heart-to-mediastinum ratio. J. Nucl. Cardiol..

[24] Verberne HJ (2005). Influence of collimator choice and simulated clinical conditions on 123I-MIBG heart/mediastinum ratios: A phantom study. Eur. J. Nucl. Med. Mol. Imaging.

[25] Nakajima K (2012). Standardization of metaiodobenzylguanidine heart to mediastinum ratio using a calibration phantom: Effects of correction on normal databases and a multicentre study. Eur. J. Nucl. Med. Mol. Imaging.

[26] Nakajima K (2014). Multicenter cross-calibration of I-123 metaiodobenzylguanidine heart-to-mediastinum ratios to overcome camera-collimator variations. J. Nucl. Cardiol..

[27] Nakajima K, Okuda K, Matsuo S, Agostini D (2016). The time has come to standardize (123)I-MIBG heart-to-mediastinum ratios including planar and SPECT methods. Eur. J. Nucl. Med. Mol. Imaging.

[28] Nakajima K, Verschure DO, Okuda K, Verberne HJ (2017). Standardization of (123)I-meta-iodobenzylguanidine myocardial sympathetic activity imaging: Phantom calibration and clinical applications. Clin. Transl. Imaging.

[29] Nakajima K, Okuda K, Verberne HJ (2019). Phase dyssynchrony and (123)I-meta-iodobenzylguanidine innervation imaging towards standardization. J. Nucl. Cardiol..

[30] Nakajima K (2017). Cardiac Sympathetic Nervous System Imaging with 123I-meta-iodobenzylguanidine. Ann. Nuclear Cardiol..

[31] Nakajima K (2017). Cross calibration of (123)I-meta-iodobenzylguanidine heart-to-mediastinum ratio with D-SPECT planogram and Anger camera. Ann. Nucl. Med..

[32] Roberts, G. *et al.* Cardiac ^123^I-MIBG normal uptake values are population-specific: Results from a cohort of controls over 60 years of age. *J. Nucl. Cardiol*. 10.1007/s12350-019-01887-6 (2019). 10.1007/s12350-019-01887-6 31529384

[33] Verschure DO (2018). A European myocardial (123)I-mIBG cross-calibration phantom study. J. Nucl. Cardiol..

[34] Pencina MJ, D’Agostino RB, D’Agostino RB, Vasan RS (2008). Evaluating the added predictive ability of a new marker: From area under the ROC curve to reclassification and beyond. Stat. Med..

[35] Motomura N (1999). Practical compensation method of downscattered component due to high energy photon in 123I imaging. Kaku Igaku.

[36] Kobayashi H (2003). Scatter correction by two-window method standardizes cardiac I-123 MIBG uptake in various gamma camera systems. Ann. Nucl. Med..

[37] Ichihara T, Ogawa K, Motomura N, Kubo A, Hashimoto S (1993). Compton scatter compensation using the triple-energy window method for single- and dual-isotope SPECT. J. Nucl. Med..

[38] Matsuo S (2009). Standardization of the heart-to-mediastinum ratio of 123I-labelled-metaiodobenzylguanidine uptake using the dual energy window method: feasibility of correction with different camera-collimator combinations. Eur. J. Nucl. Med. Mol. Imaging.

[39] Inoue Y (2013). Acquisition protocols and correction methods for estimation of the heart-to-mediastinum ratio in ^123^I-metaiodobenzylguanidine cardiac sympathetic imaging. J. Nucl. Med..

[40] Inoue Y (2017). Correction of collimator-dependent differences in the heart-to-mediastinum ratio in (123)I-metaiodobenzylguanidine cardiac sympathetic imaging: Determination of conversion equations using point-source imaging. J. Nucl. Cardiol..

[41] Hashimoto J (2017). A novel method for measuring heart-to-mediastinum ratio in ^123^I-MIBG scintigraphy using image fusion techniques. Ann. Nuclear Cardiol..

[42] Ljungberg M, Strand SE (1989). A Monte Carlo program for the simulation of scintillation camera characteristics. Comput. Methods Programs Biomed..

[43] Nakajima K (2010). Normal values for nuclear cardiology: Japanese databases for myocardial perfusion, fatty acid and sympathetic imaging and left ventricular function. Ann. Nucl. Med..

[44] Nakajima K (2007). Creation and characterization of Japanese standards for myocardial perfusion SPECT: Database from the Japanese Society of Nuclear Medicine Working Group. Ann. Nucl. Med..

[45] Nakajima K (2016). Normal values and standardization of parameters in nuclear cardiology: Japanese Society of Nuclear Medicine working group database. Ann. Nucl. Med..

