# Supplementary material for: Calibrated scintigraphic imaging procedures improve quantitative assessment of the cardiac sympathetic nerve activity
Source: Sci Rep. 2020 Dec 14;10:21834. doi: 10.1038/s41598-020-78917-8 (PMC7736873; doi:10.1038/s41598-020-78917-8)
Supplement: Supplementary file 1 — Supplementary Information. [file 41598_2020_78917_MOESM1_ESM.docx]

Supplementary information

**Calibrated scintigraphic imaging procedures improve quantitative assessment of the cardiac sympathetic nerve activity.**

*Koichi Okuda^1^, *Kenichi Nakajima^2^, Chiemi Kitamura^3^, Yumiko Kirihara^3^,
Mitsumasa Hashimoto^1^, Seigo Kinuya^4^

Supplementary Table 1. Average multicenter conversion coefficients obtained from both 705 and 1459 image sets in ^123^I-MIBG phantom image database.

|  | ^123^I-MIBG phantom image database | |  | |
| --- | --- | --- | --- | --- |
|  | 309 institutions 705 image sets | 593 institutions 1,459 image sets | | *p* value |
| CHR  LEHR  LEGP  ELEGP  LMEGP  MEGP  MELP | 0.545 ± 0.0268 (n = 21) 0.545 ± 0.0414 (n = 168)  0.631 ± 0.0455 (n = 57)  0.745 ± 0.0268 (n = 149)  0.823 ± 0.0437 (n = 102)  0.879 ± 0.0429 (n = 179) 0.894 ± 0.0349 (n = 29) | 0.548 ± 0.0210 (n = 48) 0.544 ± 0.0426 (n = 378)  0.626 ± 0.0577 (n = 90)  0.746 ± 0.0286 (n = 162)  0.830 ± 0.0493 (n = 399)  0.878 ± 0.0490 (n = 286) 0.926 ± 0.0491 (n = 96) | | n.s.  n.s.  n.s.  n.s.  n.s.  n.s.  0.0009 |

Abbreviations: *CHR*, cardiac high-resolution; *ELEGP*, extended low-energy general-purpose; *LEGP*, low-energy general-purpose; *LEHR*, low-energy high-resolution; *LMEGP*, low-medium-energy general-purpose; *MEGP*, medium-energy general-purpose; *MELP*, ME low-penetration.

Supplementary Table 2. Average multicenter conversion coefficients for combinations of gamma cameras and collimators obtained from 1459 image sets.

|  | CHR | LEHR | LEGP | ELEGP | LMEGP | MEGP | MELP |
| --- | --- | --- | --- | --- | --- | --- | --- |
| **ADAC**  Forte  **GE**  Discovery, Optima  Infinia  Millennium MG  Millennium VG  **PHILIPS**  BrightView  **PICKER**  PRISM  **SIEMENS**  e.cam, Symbia  Evo Excel, Intevo  **TOSHIBA**  e.cam, Symbia  GCA 7100/7200  GCA 9300 | -  -  -  -  -  0.55  -  -  -  -  -  - | 0.55  0.53  0.56  0.57  0.51  -  0.58  0.53  0.52  0.56  0.55  0.52 | -  -  -  0.65  0.62  -  0.62  -  -  -  -  - | -  0.74  0.75  -  -  -  -  -  -  -  -  - | -  -  -  -  -  -  -  0.84  0.83  0.83  0.79  0.72 | 0.87  0.88  0.89  0.89  0.88  0.86  0.88  0.88  0.88  0.89  - | -  -  -  -  -  -  -  0.92  0.90  0.94  -  - |

Abbreviations: *CHR*, cardiac high-resolution; *ELEGP*, extended low-energy general-purpose; *LEGP*, low-energy general-purpose; *LEHR*, low-energy high-resolution; *LMEGP*, low-medium-energy general-purpose; *MEGP*, medium-energy general-purpose; *MELP*, ME low-penetration.

Supplementary Table 3. HMR without and with multicenter conversion coefficient correction for individual heart failure patients.

| Patient | Early HMR | |  | Delayed HMR | |
| --- | --- | --- | --- | --- | --- |
|  | Uncorrected | Corrected |  | Uncorrected | Corrected |
| 1 | 1.39 | 1.41 |  | 1.33 | 1.35 |
| 2 | 1.42 | 1.44 |  | 1.14 | 1.15 |
| 3 | 1.47 | 1.49 |  | 1.20 | 1.21 |
| 4 | 1.91 | 1.96 |  | 1.29 | 1.31 |
| 5 | 1.95 | 2.00 |  | 1.55 | 1.57 |
| 6 | 1.96 | 2.01 |  | 1.89 | 1.93 |
| 7 | 2.00 | 2.05 |  | 1.83 | 1.88 |
| 8 | 2.13 | 2.18 |  | 1.92 | 1.96 |
| 9 | 2.17 | 2.23 |  | 1.88 | 1.92 |
| 10 | 2.32 | 2.38 |  | 1.69 | 1.72 |
| 11 | 2.38 | 2.45 |  | 1.89 | 1.93 |
| 12 | 2.38 | 2.45 |  | 2.42 | 2.49 |

Abbreviations: *HMR*, heart-to-mediatstinum ratio.

Supplementary Table 4. Average multicenter conversion coefficients for combinations of gamma cameras and collimators in the energy-window setting of 159 keV ± 7.5%

|  | CHR | LEHR | LEGP | ELEGP | LMEGP | MEGP | MELP |
| --- | --- | --- | --- | --- | --- | --- | --- |
| **SIEMENS**  e.cam, Symbia | - | 0.55 | 0.65 | - | 0.85 | 0.90 | 0.95 |

Abbreviations: *CHR*, cardiac high-resolution; *ELEGP*, extended low-energy general-purpose; *LEGP*, low-energy general-purpose; *LEHR*, low-energy high-resolution; *LMEGP*, low-medium-energy general-purpose; *MEGP*, medium-energy general-purpose; *MELP*, ME low-penetration.


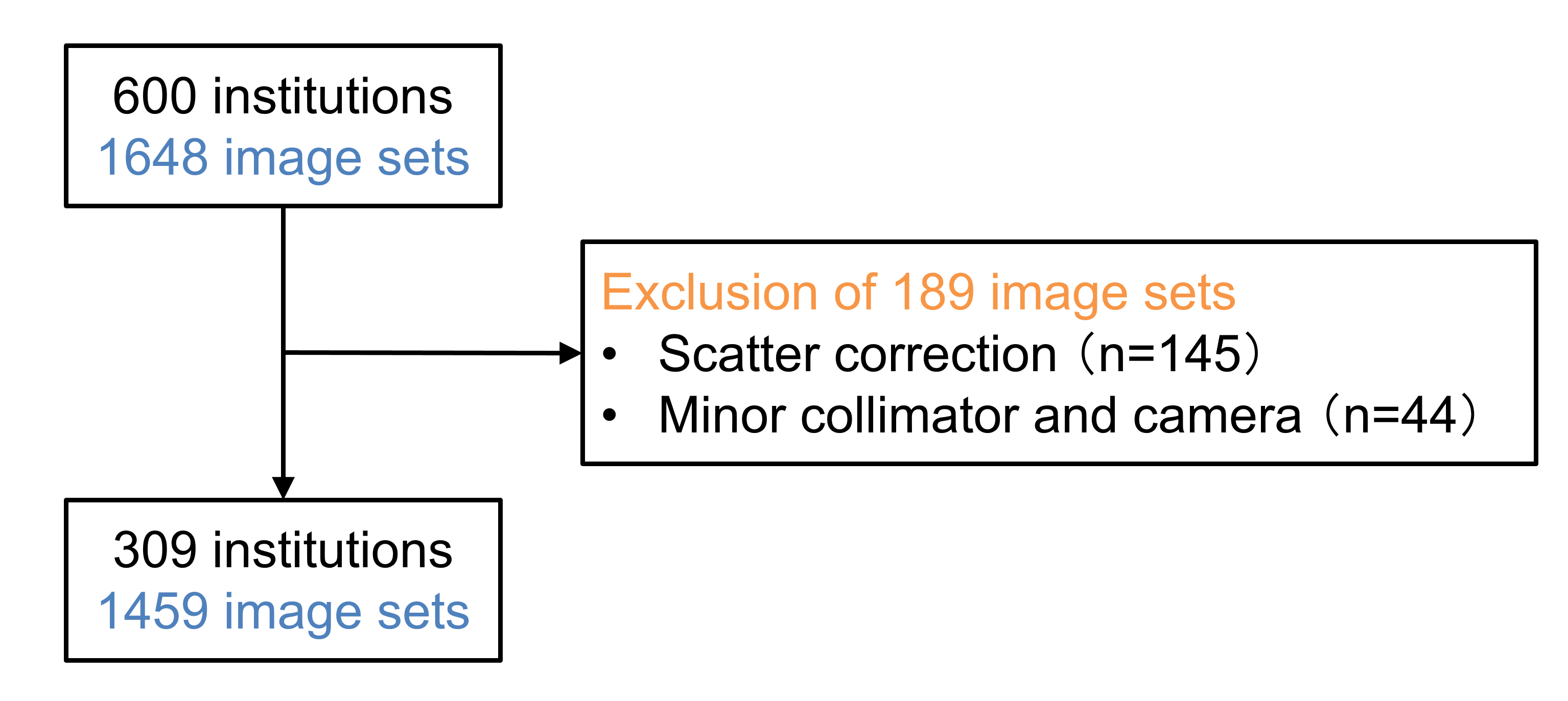


Supplementary Figure 1. Multicenter ^123^I-MIBG phantom image datasets selected based on two exclusion criteria. Selected 1459 image sets acquired with various energy-window settings for ^123^I-MIBG and Imaging matrices of 64×64, 128×128, 256×256, and 512×512 were included in this ^123^I-MIBG phantom image database.
